# Ultrasonographic Pattern of Testicular Metastasis of Clear Cell Renal Cell Carcinoma with Pathological Correlation

**DOI:** 10.5334/jbr-btr.937

**Published:** 2016-03-31

**Authors:** Florent Libert, Mathieu Cabri-Wiltzer, Emmanuel Dardenne, Anne-Philippe Draguet, Thierry Puttemans

**Affiliations:** Department of Radiology, Clinique Saint-Pierre, Ottignies, Belgium; Department of Urology, Clinique Saint-Pierre, Ottignies, Belgium; Department of Pathology, IPG, Gosselies, Belgium

**Keywords:** Testicular metastasis, Clear cell renal cell carcinoma, Ultrasonography

## Abstract

Two cases of testicular metastases of a clear cell renal cell carcinoma sharing a very similar ultrasonographic pattern are reported. The observed pattern – masses containing multiple tiny cyst-like areas – is very similar to that of a previously described ovarian metastasis of clear cell renal parenchymal tumor and can be explained by histopathologic features. Despite the small number of cases, this ultrasonographic pattern of testicular mass may be specific for metastasis of clear cell renal cell carcinoma origin.

## Introduction

Only few ultrasonographic descriptions of testicular metastases have been reported in the literature. In textbooks, their sonographic appearance is usually described as solid hypoechoic lesions indistinguishable from other types of tumor [1]. The majority of these reports focus on pathologic pattern or on CT aspect without precise ultrasonographic description [2,3]. Herein, two cases of testicular metastases of clear cell renal cell carcinoma (CCRCC) are presented, with very similar ultrasonographic patterns to that of a previously described ovarian metastasis of the same primary tumor [4]. This ultrasonographic pattern therefore is highly suggestive of metastases of CCRCC origin.

## Case Reports

### Case 1

A 69-year-old man without relevant medical history presented with a small lump in the right testis. Ultrasound examination revealed a solitary intra-testicular hyperechoic 12 mm tumor with heterogeneous appearance and with multiple small cystic-like areas (Figure 1A). On colour-Doppler ultrasound, the tumor was hypervascular compared to adjacent parenchyma (Figure 1B). Dosage of α-fetoprotein, human chorionic gonadotropin and lactate dehydrogenase serum markers was normal. Computed tomography (CT) revealed a hypervascular parenchymal tumor in the right kidney. The patient underwent a right radical nephrectomy as well as a right inguinal orchiectomy. Histopathological and immunohistochemical examination demonstrated a primary renal CCRCC metastasized to the testis (Figure 1C), staged pT1bN0M1.

**Figure 1A:**
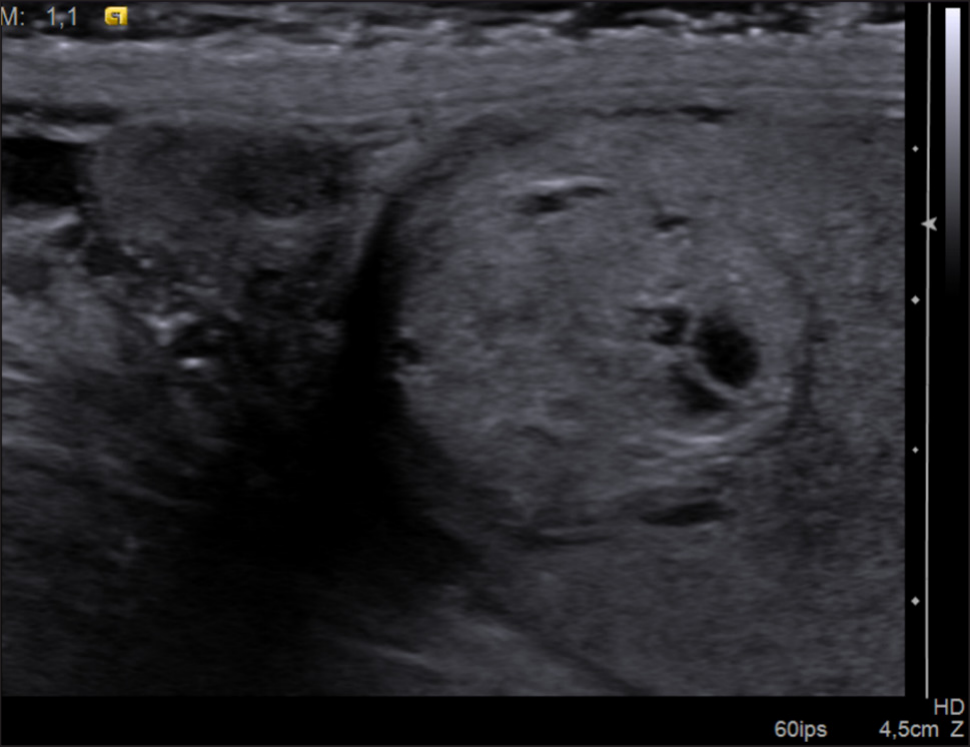
Right testicular CCRCC metastasis in a 69-year-old man. Longitudinal US of the right testicular upper pole showing a slightly heterogeneous and hyperechoic tumor, containing multiple anechoic areas and bounded by a non-continuous hypoechoic halo.

**Figure 1B:**
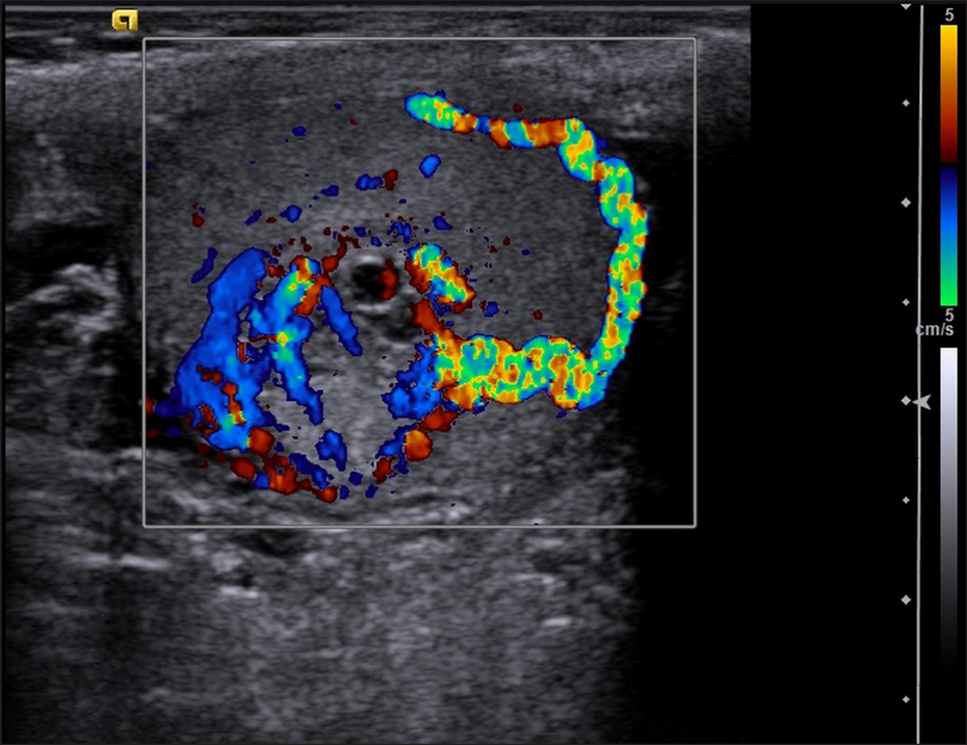
Colour Doppler ultrasound demonstrates tumoral hypervascularisation.

**Figure 1C:**
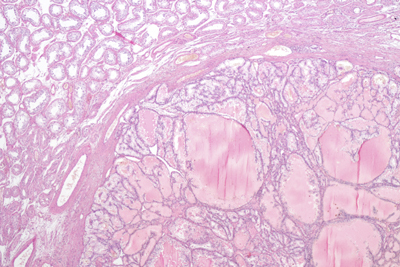
Pathological examination. Photomicrograph in the periphery of the tumor showing a multi-cystic lesion delineated by a fibrous capsule. Cystic appearance is due to lumen filling with eosinophilic fluid and cellular debris (hematoxylin and eosin stain; original magnification x50).

### Case 2

**Figure 2:**
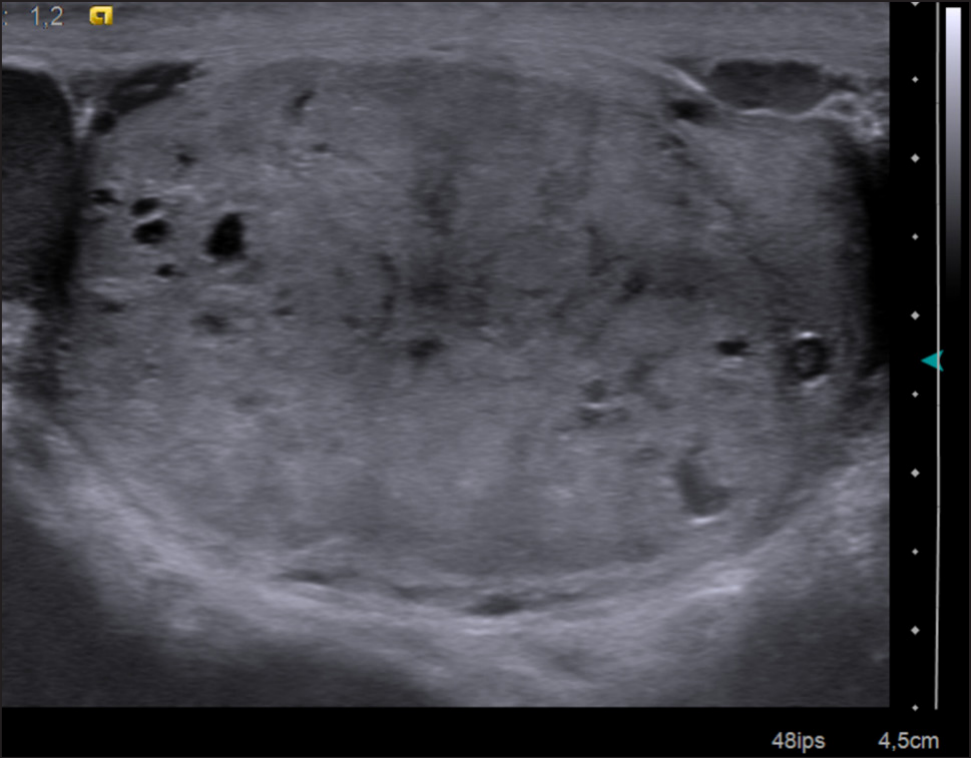
Left testicular CCRCC metastasis in a 77 year-old man. Longitudinal US of the left testis showing an intra-testicular slightly hyperechoic tumor replacing almost the entire testis. Solid component is dominant but several cystic areas are also depicted. Note the similarities with Figure 1A.

A 77-year-old man presented with painless swelling of the left hemiscrotum. He underwent a partial left nephrectomy for a CCRCC five years earlier and later developed pulmonary metastases. On physical examination, there was a firm left testicular mandarin-sized mass. Scrotal sonography showed a hyperechoic intra-testicular mass (diameter 47 mm) replacing almost the entire left testis. This heterogeneous mass contained multiple small anechoic cystic-like areas (Figure 2) and was hypervascular on color-Doppler. Serum tumor markers were within normal limits. The metastatic nature of this testicular mass was confirmed by histological examination of the orchiectomy specimen.

## Discussion

The testis is an uncommon site for metastases, with a reported prevalence ranging between 0.02% [5] and 0.06% [6] at autopsy in the general oncologic population. The most common primary tumor sites are the prostate, kidney, lung, colon and skin [3,7]. Though rare, metastases account for nearly 10% of testicular tumors in patients over 60 years of age [8]. In the majority of cases, a known oncologic context supports the diagnosis since testicular metastases usually occur in cases of advanced multifocal metastatic neoplasia [9]. Yet it may occasionally be the presenting symptom of malignant disease and thus become challenging [3]. Testicular metastases must be suspected in case of a testicular mass in an elderly male, in particular with normal serum alpha-fetoprotein and human chorionic gonadotropin levels. The differential diagnosis of a testicular mass in the elderly mainly includes lymphoma, accounting for one-third of testicular tumors in patients over 60 years, and germ cells tumors (about 20%), including spermatocytic seminoma (2%), a subtype with good prognosis and more frequent in elderly men [8].

Since ultrasonography has shown a high sensitivity in tumor detection (nearly 100%) and in discrimination of intratesticular from extratesticular localization (97–99%) [10,11,12], ultrasound is the modality of choice in the initial work-up of scrotal swellings. In general and with rare exceptions only, a solid intra-testicular lesion must be considered malignant. Sonographic characterization of the histological nature however remains limited largely because of overlap of sonographic features [9].

The features of the two cases of testicular metastases of CCRCC are almost similar to sonographic pattern of an ovarian metastasis of a renal clear cell carcinoma previously described in the literature [4]. In all three cases, the solitary lesion was incompletely delineated by a hypoechoic rim of variable thickness. These lesions appeared heterogeneous and hyperechoic, containing multiple cystic-like areas, and were hypervascular at the color-Doppler study.

This sonographic pattern reflects the gross morphology and underlying histologic characteristics of CCRCC (primary tumor as well as their metastases) which can be considered as quite specific. CCRCC commonly presents as a well-circumscribed mass with a capsule or pseudocapsule. Typically, it is characterized by solid nests and hollow tubules composed of polygonal epithelial cells with clear and abundant cytoplasm. The transparency of the cytoplasm results from intra-cytoplasmic accumulation of droplets of glycogen and lipids, giving rise to the yellow coloration at macroscopic examination and probably to increased echogenicity due to multiple intra-cellular interfaces. The tumor may also show cystic areas when luminal spaces of the tubular structures dilate and become filled with fluid, necrotic material and red blood cells, explaining the cystic areas on ultrasonography. Cellular nests and tubules are separated by a delicate branching network of highly vascularized connective tissue.

Based on this sonographical-pathological correlation, facing a testicular lesion with these characteristics in an elderly male should raise suspicion of a metastases of CCRCC.

## Competing Interests

The authors declare that they have no competing interests.
